# Notes and News

**Published:** 1952-01

**Authors:** 


### Notes and News

### THE ROYAL COLLEGES

Professor C. Bruce Perry has been elected to the council of the Royal College of Physicians.

*M.R.C.P.* Bowden, D. H. Fernley M. (Gloucester Royal Hospital).

*F.R.C.S.(Eng.).* Bolt, D. E. Levey, A. H. Slade, N. Soltan, D. H. K.

*M.R.C.S., L.R.C.P. (Conjoint Board).* Arney, K. B. Tricks, N. C.

### UNIVERSITY OF BRISTOL

*Bristol Royal Hospital Board's Gold Medal,* Lloyd, June K.

*Bristol Royal Hospital Board's Silver Medal,* Benson, W. G.

*Dental Gold Medal,* Chivers, A. H.

*Degrees of M.B., Ch.B.(Brist.)*

*December* 1951

Barwick, C.

Blake, Audrey M.

Catford, G. V.

Haines, Ann M.

Hillier, G. J.

Hunt, R. J.

Iles, R. A.

Jarvis, D. J. A.

Joyce, M. H. B.

Lawson, June P.

Macleod, Denise G.

Motton, Mary.

Murison, I. C.

Norris, P.

Peacock, Gillian F.

Rowland, A. J.

Sartori, N.

Sheldrick, M.

Sidorowicz, Antonina J.

Tricks, N. C.

Trump, D. W.

Wright, D. W.

### YEOVIL DISTRICT HOSPITAL

The new Out-patient Department is completed and is already in use. In addition to the Clinics already established Mr. T. Price, Orthopaedic Surgeon, will be holding Out-patient Clinics here.

Mr. D. Mearns Milne has been appointed Thoracic Surgeon to the South Somerset Clinical Area and a Thoracic Out-patient Clinic will be commencing later in the year.

